# Reactivation of IgG-switched memory B cells by BCR-intrinsic signal amplification promotes IgG antibody production

**DOI:** 10.1038/ncomms9575

**Published:** 2015-10-13

**Authors:** Johannes Lutz, Kai Dittmann, Michael R Bösl, Thomas H Winkler, Jürgen Wienands, Niklas Engels

**Affiliations:** 1 Institute of Cellular and Molecular Immunology, Georg-August-University of Göttingen, Medical Faculty, Humboldtallee 34, 37073 Göttingen, Germany; 2 Max Planck Institute of Neurobiology, Transgenic Core Facility, 82152 Martinsried, Germany; 3 Hematopoiesis Unit, Department of Biology, Nikolaus-Fiebiger-Center for Molecular Medicine, Friedrich-Alexander-University Erlangen-Nürnberg, Glückstrasse 6, 91054 Erlangen, Germany

## Abstract

Secondary antibody responses are marked by faster kinetics, improved antibody affinity and a switch from IgM to other immunoglobulin isotypes, most notably IgG, compared with primary responses. These changes protect from reinfection and represent the principle of most vaccination strategies. Yet, the molecular mechanisms that underlie B-cell memory responses are unclear. Here we show, by inactivating the immunoglobulin tail tyrosine (ITT) signalling motif of membrane-bound IgG1 in the mouse, that the ITT facilitates maintenance and reactivation of IgG-switched memory B cells *in vivo*. The ITT motif equips IgG-switched cells with enhanced BCR signalling capacity, which supports their competitiveness in secondary immune reactions and drives the formation of IgG-secreting plasma cells even in the absence of T-cell help. Our results demonstrate that ITT signalling promotes the vigorous production of IgG antibodies and thus provide a molecular basis for humoral immunological memory.

## Abstract (web summary)

Antigen receptors on memory B cells enhance their signaling strength by recruiting the cytosolic Grb2 adaptor to their ITT phosphorylation motifs. Here the authors show that inactivating the ITT motif of mouse mIgG1 impairs IgG1 production and T-cell independent reactivation of memory B cells.

## Graphical abstract

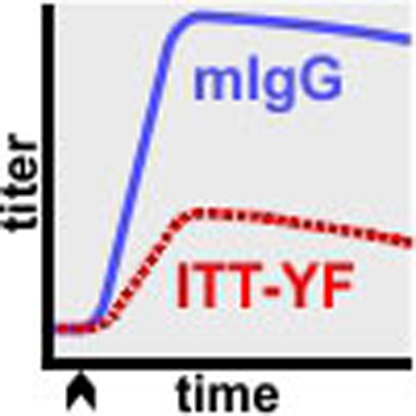

The production of antibodies by B lymphocytes is initiated on recognition of extracellular antigen by clonotypic antigen receptors expressed on the B-cell surface. On secondary antigen encounter, antibody responses are more vigorous and dominated by the production of immunoglobulin G (IgG) antibodies. However, the mechanisms that encode such improved memory responses remain unclear. Binding of antigen to B-cell antigen receptors (BCRs) initiates a complex series of signalling events that, in combination with costimulatory signals, promote cellular activation and differentiation into antibody-producing plasma cells^1^. A monomeric BCR unit is composed of a membrane-bound immunoglobulin (mIg) molecule of one of the five classes, μm, δm, γm, ɛm, or αm, that non-covalently associates in a 1:1 stoichiometry with a heterodimer of mIg-associated α and β proteins (Igα/β, CD79a/b) on the cell surface^2^,^3^. Antigen-mediated reorganization of BCR complexes triggers the activation of cytoplasmic protein tyrosine kinases (PTKs) that phosphorylate immunoreceptor tyrosine-based activation motifs (ITAMs) in the cytoplasmic domains of Igα and Igβ (refs 4, 5, 6, 7). Phosphorylated ITAM tyrosines serve as docking sites as well as allosteric activators for PTKs of the spleen tyrosine kinase (Syk) family^8^,^9^. In addition, Igα contains an evolutionary conserved non-ITAM tyrosine residue (Y204) that on phosphorylation recruits the central adaptor protein Src-homology 2 (SH2) domain containing adaptor protein of 65 kDa (SLP65, also called BLNK) via its SH2 domain^10^. Syk-mediated phosphorylation of SLP65 then enables the formation of a supramolecular signalling platform from which the activation of ubiquitous signalling cascades is initiated^11^.

The importance of individual ITAM and non-ITAM tyrosine residues in Igα and Igβ for the development of the B-cell compartment and antibody production has been analysed in a set of mouse strains expressing tyrosine-to-phenylalanine (YF) mutant versions of Igα or tyrosine-to-alanine mutant Igβ (refs 12, 13, 14). Inactivation of either ITAM motif alone has little impact on B-cell development and overall antibody production. Combined inactivation of both BCR ITAMs, however, causes an almost complete block in B-cell development at the proB- to preB-cell transition^13^. The SLP65-binding non-ITAM tyrosine motif in Igα is dispensable for the development of B2 B cells but has a specific function in supporting T-independent antibody responses^14^.

Immunization with T-dependent antigens initiates the formation of germinal centres in which activated B cells can undergo affinity maturation and immunoglobulin class switching. The cellular outputs of germinal centres are antibody-secreting plasma cells and memory B cells that are poised to differentiate into plasma cells on antigen reencounter^15^. Immunoglobulin class-switch recombination to IgG or IgE isotypes equips B cells with altered BCRs that in addition to the canonical Igα/β signalling subunit contain signalling motifs in the cytoplasmic domains of the respective mIg heavy chains, which are absent in mIgM- and mIgD-BCRs of naive cells. On antigen binding, these immunoglobulin tail tyrosine (ITT) motifs become phosphorylated and serve as docking sites for the ubiquitous cytoplasmic adaptor protein growth factor receptor-bound 2 (Grb2). Recruitment of Grb2 and its binding partner Bruton's tyrosine kinase (Btk) into the signalosome of mIgG- and mIgE-containing BCRs amplifies BCR-proximal signalling events, including phosphorylation of SLP65 and subsequent Ca^2+^ mobilization^16^,^17^,^18^. The essential importance of the entire cytoplasmic domains of mIgG and mIgE for antibody responses of the respective isotype was demonstrated in genetically engineered mouse models^19^,^20^,^21^. Whether or not the ITT motif is responsible for the function of the cytoplasmic tails of mIgG and mIgE *in vivo*, remained, however, unknown.

Here we demonstrate that inactivation of the ITT by a YF substitution in mIgG1 leads to substantially impaired IgG1 antibody responses in the mouse. This is due to the reduced generation of IgG1-secreting plasma cells despite normal memory cell formation. In heterozygous animals, cells expressing YF-mutant mIgG1 have a strong competitive disadvantage in entering the plasma cell compartment compared with cells expressing wild-type mIgG1. Furthermore, the ITT improves the reactivation of memory B cells in a T-cell-independent manner. Our results show that ITT signalling is essential for B-cell memory *in vivo*.

## Results

### ITT phosphorylation enhances BCR-proximal signalling

**Figure 1:**
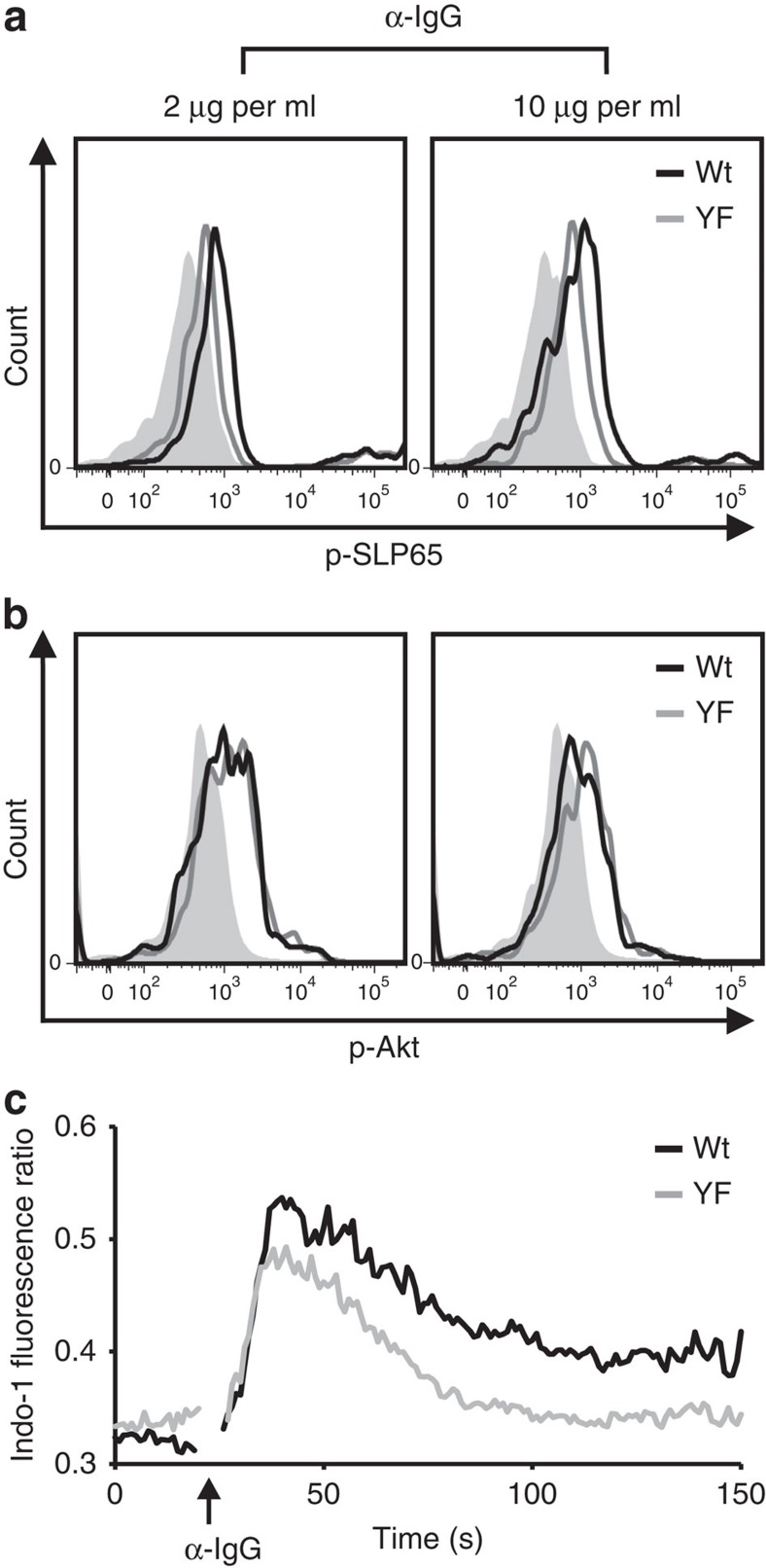
The ITT amplifies BCR-induced Ca^2+^ signalling. Splenic B cells from naive wild-type (wt) and ITT-mutant (YF) mice were isolated by magnetic cell sorting and stimulated with either low (2 μg ml^−1^, left panels) or high (10 μg ml^−1^, right panels) doses of anti-IgG F(ab')_2_ fragments. Unstimulated cells served as controls (filled histograms). Subsequently, cells were stained with phospho-specific antibodies to either SLP65 (**a**) or Akt (**b**) wt: (black curves), YF: mIgG1-YF (grey curves). (**c**) Splenic B cells from three age-matched male mice of each genotype that had been immunized with sheep red blood cells for 11 days were pooled, loaded with Indo-1-AM and stained with FITC-labeled monomeric Fab fragments against IgG1. Ca^2+^ mobilization of mIgG1-positive cells was monitored before and after stimulation with anti-IgG F(ab')_2_ fragments (20 μg ml^−1^, indicated by an upward arrow) in the presence of 1 mM extracellular CaCl_2_. Data are representative of three to four experiments.

To investigate the function of the ITT signalling motif *in vivo*, we introduced a point mutation into the genome of C57BL/6 mice that causes a YF substitution in the cytoplasmic domain of mIgG1 (mIgG1-YF; for details see Supplementary Figs 1 and 2). First, we compared the signalling capabilities of YF-mutant and wild-type mIgG1-BCRs in primary cells and used phosphorylation of the adaptor protein SLP65 and the serine/threonine kinase v-akt murine thymoma viral oncogene homologue 1 (Akt, also called protein kinase B, PKB) as readouts for the activation of BCR-regulated signalling pathways (Fig. 1a,b, respectively, and Supplementary Fig. 3A). In line with our previous observations in transfected B cells^16^,^17^ inactivation of the ITT caused a substantial reduction in phosphorylation of SLP65 under optimal and suboptimal stimulation conditions (Fig. 1a). By contrast, the activation of Akt was not affected by the ITT mutation (Fig. 1b), showing that the ITT specifically improves the activation of Ca^2+^-regulating components in IgG-switched cells. As control, splenic B cells of both genotypes were treated with F(ab')_2_ fragments to IgM, which demonstrated their equal signalling capacities (Supplementary Fig. 3B). Next, we tested whether the enhanced phosphorylation of SLP65 was associated with augmented Ca^2+^ mobilization in IgG1-switched B cells. To this end, purified splenic B cells from immunized mice were loaded with Indo-1 and stained with FITC-conjugated non-activating monomeric anti-IgG1 Fab fragments. Subsequently, mIgG–BCRs were stimulated with polyclonal anti-IgG F(ab')_2_ fragments. This setting allowed us to monitor BCR-induced Ca^2+^ mobilization selectively in mIgG1-positive cells (Fig. 1c and Supplementary Fig. 4A). Inactivation of the ITT caused a strong reduction in BCR-induced Ca^2+^ mobilization as previously shown in transfected B cells. As control, splenic B cells of both genotypes were treated with F(ab')_2_ fragments to IgM (Supplementary Fig. 4B). In conclusion, phosphorylation of the ITT motif greatly improves mIgG–BCR signalling by promoting BCR-induced Ca^2+^ signalling even under conditions of suboptimal stimulation. This improved reactivity of mIgG–BCRs is brought about by the recruitment of a Grb2/Btk signalling module to the ITT motif, which facilitates activation of phospholipase C-γ and subsequent second messenger generation^16^,^17^.

### Germinal centre B cells do not require ITT signalling

**Figure 2:**
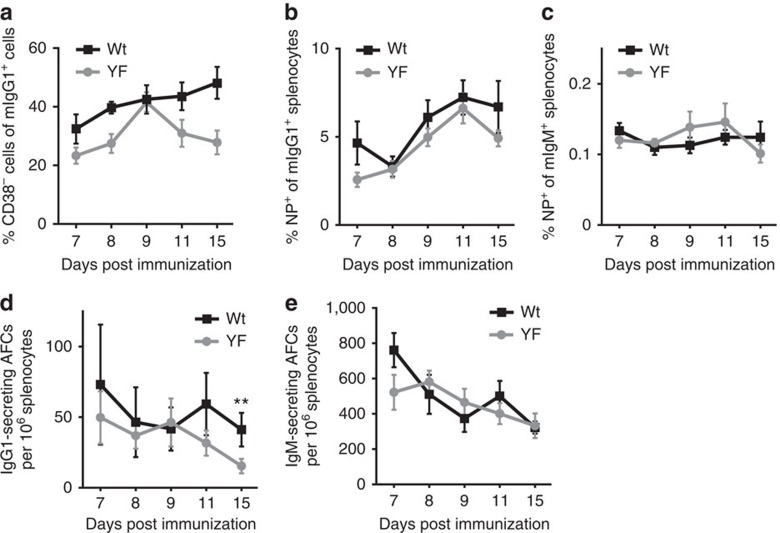
IgG-switched germinal centre B cells do not require ITT signalling. Cohorts of sex- and age-matched male and female wild-type (wt) and ITT-mutant (YF) mice were immunized with NP–KLH. (**a**) Kinetics of germinal centre B-cell generation in the spleen. Relative frequency of B220-positive, CD38-negative cells among mIgG1-expressing cells during the course of the experiment. (**b**) NP-specific splenic B cells were identified with NP8-PE. The percentage of NP-binding mIgG1-positive B cells is shown. (**c**) Kinetics of mIgM-positive, NP-binding splenic B cells. (**d**,**e**) The numbers of antibody forming cells (AFCs) per 10^6^ splenocytes was determined by ELISPOT assays for IgG1 (**d**) and IgM (**e**). Numbers of analysed animals are as follows: **a**,**b**,**c** and **e** shows mean values±s.e.m. of three independent experiments with three animals per genotype and time point (that is, *n*=9 per time point and genotype). **d** shows data of two independent experiments (that is, *n*=6 per time point and genotype). Statistical significance was determined by Mann–Whitney test. **P<0.01.

Membrane-bound IgG-containing BCRs are expressed for the first time in the life of a B cell following successful immunoglobulin class-switch recombination, which predominantly occurs during germinal centre reactions. To test whether B cells require ITT signals to prevail in the competitive environment of a germinal centre, we investigated the cellular composition of germinal centres in mIgG1-YF mice during a primary immune response against the hapten 4-hydroxy-3-nitrophenylacetyl (NP) coupled to the carrier protein keyhole limpet haemocyanin (KLH). We followed the generation of mIgG1-expressing cells during the first 15 days of the primary response and used CD38 as a marker to differentiate between memory (CD38-positive) cells and activated germinal centre (CD38-negative) B cells^22^ (Supplementary Fig. 5). The kinetics of mIgG1-positive germinal centre B-cell generation during the primary response was very similar in mIgG1-YF and wild-type mice, even though there was a trend towards a somewhat higher production in wild-type mice at days 11 and 15 (Fig. 2a). Also, mIgG1-expressing cells specific for the hapten NP were produced in similar amounts in both genotypes (Fig. 2b and Supplementary Fig. 6) as were NP-specific mIgM-expressing cells (Fig. 2c). Furthermore, we analysed the generation of antibody-secreting cells during the primary response to NP–KLH. The numbers of IgG1-secreting cells in the spleens of both genotypes were comparable during the first 9 days of the primary antibody response. After that period the numbers of IgG1-producing cells in wild-type animals were two- to threefold higher than in mIgG1-YF animals (Fig. 2d). The numbers of IgM-secreting cells were the same in both animal groups throughout the experiment (Fig. 2e). Thus, ITT signalling does not seem to be a major driving force for the generation of IgG-switched B cells in the highly selective environment of a germinal centre but seems to favour the formation of IgG-secreting cells already in the primary immune response.

### The ITT improves the maintenance of IgG-switched B cells

**Figure 3:**
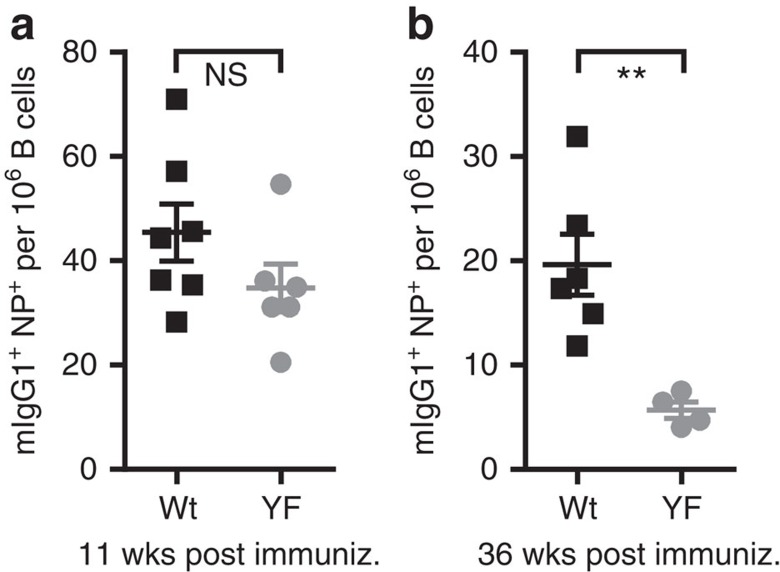
The ITT improves the maintenance of IgG-switched memory B cells. (**a**) Frequencies of mIgG1+, NP-specific B cells in spleens of male wild-type (wt, *n*=7) and ITT-mutant (YF, *n*=6) mice 11 weeks after immunization with NP–KLH are given as number of cells per 10^6^ B220-positive cells. (**b**) Cells were analysed as in **a** 36 weeks after immunization (wt: *n*=6, YF: *n*=4). Statistical significance was determined by Mann–Whitney test. NS: not significant, *P<0.05, **P<0.01. Immuniz., immunization; wks, weeks.

Even though ITT signals were not essential for the generation of IgG-switched cells during germinal centre reactions, the possibility remained that their survival in the periphery may be regulated by the ITT. Hence, to test whether the ITT motif contributes to the maintenance of memory B cells, we determined the frequency of NP-specific mIgG1-positive B cells in the spleens of wild-type and ITT-mutant mice 11 weeks and 36 weeks after immunization with NP–KLH (Supplementary Fig. 7). Whereas the frequency of antigen-specific mIgG1-expressing cells was similar in both genotypes 11 weeks after immunization (Fig. 3a), their number was roughly four times higher in wild-type animals 36 weeks after immunization (Fig. 3b). We conclude that the presence of intact ITT motifs in the cytoplasmic domains of membrane-bound IgG molecules improves the lifespan of IgG-switched memory B cells, presumably by enhanced tonic signalling of ITT-containing mIgG–BCRs^23^.

### ITT signalling promotes production of IgG antibodies

**Figure 4:**
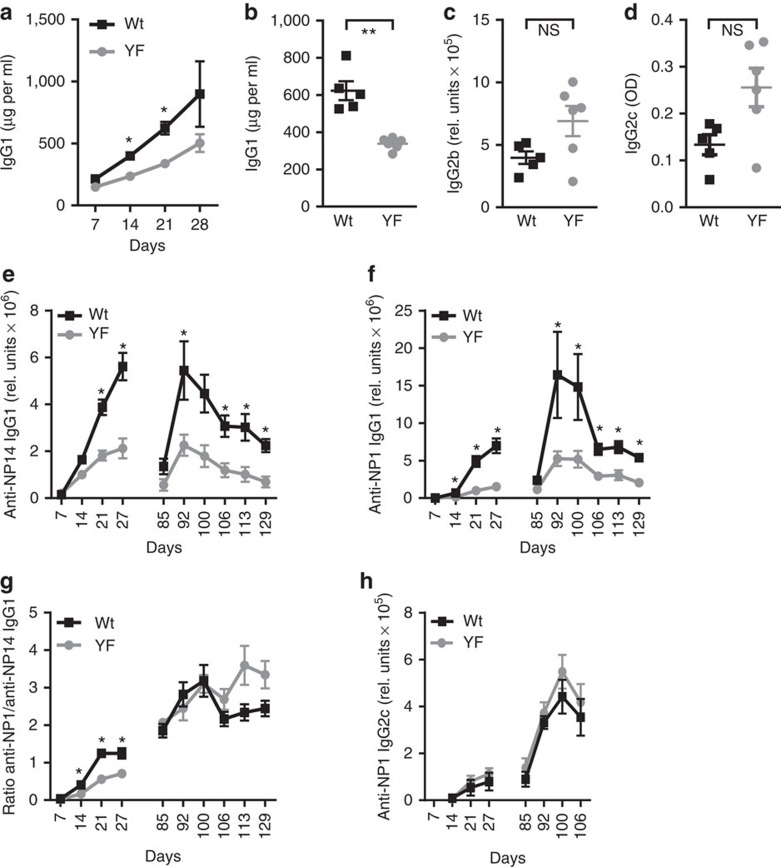
The ITT promotes IgG antibody production. (**a**) Wild-type (wt, black line) and homozygous mIgG1-YF (YF, grey line) female animals were immunized with NP–KLH in aluminium hydroxide and total IgG1 titres were monitored for 28 days. Data are shown as mean±s.e.m. of 5–6 animals per group. Titres for IgG1 (**b**), IgG2b (**c**) and IgG2c (**d**) of the animals shown in **a** were determined at day 21 after immunization. Error bars indicate mean±s.e.m. (**e**) Wild-type and mIgG1-YF male mice were immunized as in **a** and boosted 85 days later. The production of total NP-specific antibodies was determined using ELISA plates coated with NP14-BSA. Data are shown as mean of 4–5 animals per group±s.e.m. (**f**) The amount of high-affinity NP-specific antibodies in the sera of the animals shown in **e** were analysed using NP1-BSA-coated ELISA plates. (**g**) The ratio of high affinity to total NP-specific antibodies in the sera from (**e**,**f**) are shown as mean±s.e.m. (**h**) Titres of high-affinity NP-specific IgG2c antibodies in the same sera as in **e** and **f** are shown as mean±s.e.m. Statistical significance was determined by Mann–Whitney test. *P<0.05, **P<0.01. Data are representative of two independent experiments.

To investigate the influence of ITT signals on IgG production, we analysed the kinetics of IgG1 antibody formation and affinity maturation against NP in ITT-mutant and wild-type animals. Total IgG1 antibody titres in mIgG1-YF mice increased much slower than in wild-type animals (Fig. 4a), and were reduced approximately by half on day 28 of the primary response (Fig. 4b). These differences in IgG1 production during the primary response are consistent with the differences in early plasma cell formation (Fig. 2d). The isotypes IgG2b and IgG2c were moderately elevated in the sera of mIgG1-YF mice (Fig. 4c,d) indicating the existence of compensatory mechanisms as observed before^19^. Next, we compared the kinetics of total and high-affinity NP-specific IgG1 antibodies in mIgG1-YF and wild-type mice during primary and secondary immune responses. Note that re-immunization was done at a time point when numbers of antigen-specific mIgG1-positive memory B cells were roughly the same in both genotypes (Fig. 3a). Again, the production of IgG1 in ITT-mutant mice was strongly diminished compared with wild-type animals. IgG1 titres during recall responses in mIgG1-YF animals were reduced to approximately one-third of what we observed in wild-type controls and thus did not even reach the level of primary response titres of wild-type mice (Fig. 4e). Analysis of high-affinity NP-specific IgG1 antibodies gave similar results (Fig. 4f). The ratio of high-affinity to total NP-specific IgG1 titres was moderately elevated in wild-type animals during primary responses, yet increased over time in a comparable manner in both genotypes (Fig. 4g). As control, we determined the production of high-affinity IgG2c antibodies, which was similar in both genotypes (Fig. 4h). These results show that *in vivo* ITT-induced signals are essential to generate proper IgG antibody responses.

### The ITT improves the competitiveness of IgG-switched cells

**Figure 5:**
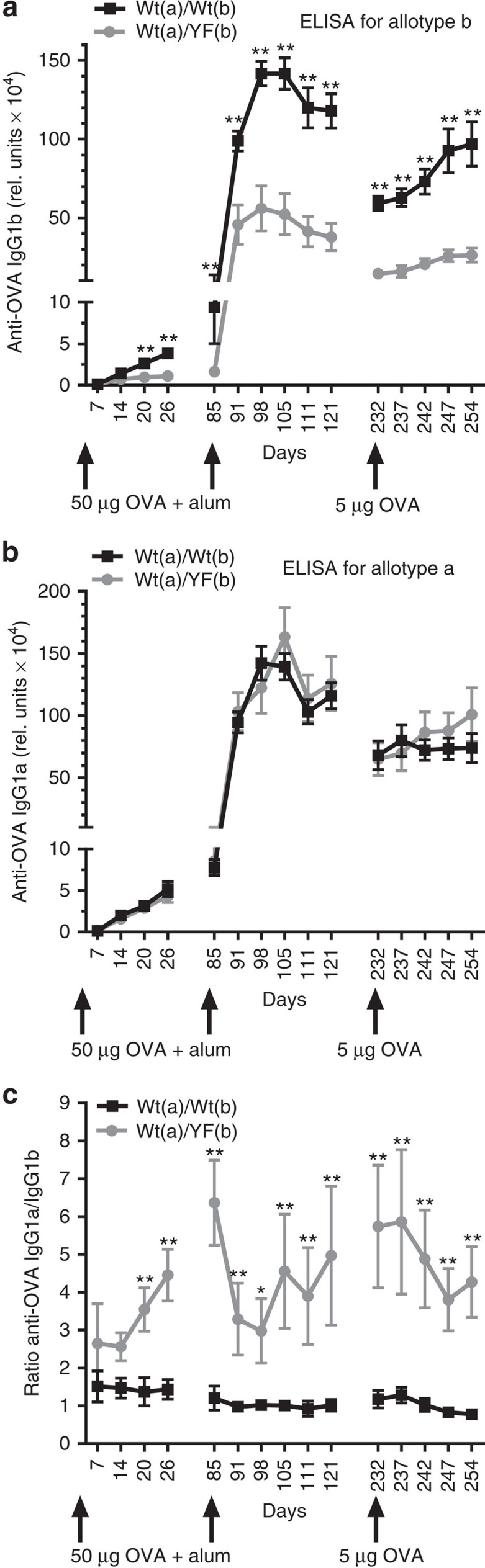
The ITT augments the competitiveness of mIgG-expressing B cells. Heterozygous male animals whose B cells expressed either wild-type mIgG1^a^ or YF-mutant mIgG1^b^ (grey lines) and control animals expressing both allotypes in wild-type configuration (black lines) were immunized with 50 μg ovalbumin in aluminium hydroxide and were boosted the same way 85 days later. On day 232, mice were challenged with 5 μg OVA (w/o alum). The production of OVA-specific IgG1^b^ (**a**) and IgG1^a^ antibodies (**b**) was determined by ELISA. Data are shown as mean±s.e.m. of 4–6 animals per group. (**c**) The ratio of IgG1^a^ to IgG1^b^ titres was calculated for each animal and is depicted as mean±s.e.m. Black line: wt/wt, grey line: wt/YF. Statistical significance was determined by Mann–Whitney test. *P<0.05, **P<0.01. Data are representative of three independent experiments.

To compare the performance of wild-type and ITT-mutant B cells directly in the same animals, we bred mIgG1-YF BL/6 mice expressing ITT-mutant mIgG1^b^ with Balb/c mice expressing wild-type mIgG1^a^. Owing to allelic exclusion, B cells of heterozygous F1 animals expressed either wild-type (allotype a) or YF-mutant (allotype b) mIgG1. Animals that had both allotypes in wild-type form served as controls. Allotype-specific analysis of IgG1 production in animals that were immunized with ovalbumin (OVA) in aluminium hydroxide (alum) showed that wild-type mIgG1^a^-expressing B cells contributed up to six times more to the production of soluble IgG1 than their mIgG1^b^-expressing ITT-mutant counterparts both during primary and secondary responses (Fig. 5a,c, grey graph). In control animals, both allotypes of IgG1 were produced in equal amounts (Fig. 5b) and, hence, were present in an almost 1:1 ratio during the entire course of the experiment (Fig. 5c, black graph). In conclusion, these results reveal that ITT-mediated signal amplification increases the competitiveness of mIgG-expressing cells, which facilitates their contribution to antibody production.

### ITT signals promote plasma cell formation

**Figure 6:**
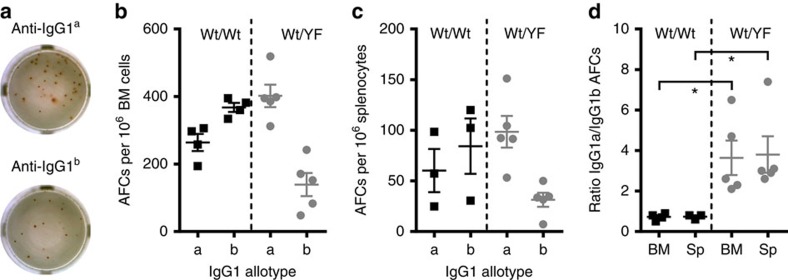
The ITT promotes plasma cell generation. Animals from Fig. 5 were sacrificed on day 22 of the recall response and the number of antibody forming cells (AFCs) was determined by ELISPOT assays. (**a**) Representative wells of IgG1^a^-producing (upper photo) and IgG1^b^-producing (lower photo) cells from the same ITT-heterozygous animal are shown. Frequencies of AFCs per one million cells from bone marrow (BM; **b**) and spleens (**c**) are shown. (**d**) The ratio of IgG1^a^ to IgG1^b^ AFCs in the spleens (Sp) and BM was calculated for each animal. Error bars represent mean±s.e.m. of three (wt/wt) and five (wt/YF) analyses, respectively. Statistical significance was determined by Mann–Whitney test. *P<0.05.

To test whether reduced antibody production by ITT-mutant B cells was linked to impaired plasma cell generation, we determined the frequency of OVA-specific IgG1 antibody forming cells in heterozygous F1 animals at day 21 of the recall response by means of allotype-specific ELISPOT assays (Fig. 6a). In accordance with the diminished IgG1^b^ serum titres (Fig. 5) we observed an approximately fourfold reduction in the number of YF-mutant IgG1^b^-producing antibody forming cells compared with IgG1^a^-producing cells both in the bone marrow (Fig. 6a,b,d) as well as in the spleens of heterozygous F1 animals (Fig. 6c,d). We conclude that ITT signalling supports the generation of plasma cells from the memory cell compartment.

### ITTs promote T-cell-independent activation of memory B cells

**Figure 7:**
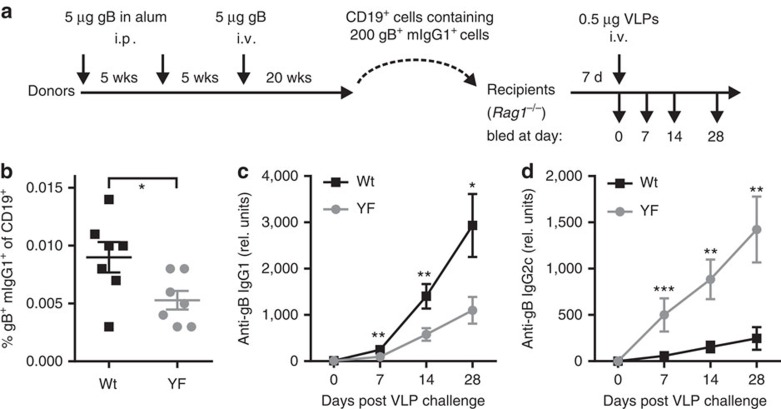
The ITT promotes T-cell-independent reactivation of IgG-switched memory B cells. (**a**) Experimental outline. Female wild-type and ITT-mutant mice (seven animals per genotype) were repeatedly immunized with purified gB with or without alum as specified at the indicated time points. Splenocytes depleted of T cells and purified with anti-CD19 magnetic beads were adjusted to contain 200 mIgG1-positive, gB-specific cells and were injected into 10 *Rag1*-deficient mice. Seven days later recipient animals were challenged with 0.5 μg of gB-containing virus-like particles (VLPs) and sera were collected at the indicated time points. (**b**) Frequencies of gB-binding mIgG1-positive B cells in donor mice before transfer. Titres of gB-specific IgG1 (**c**) and IgG2c (**d**) in sera of *Rag1*-deficient recipient mice after challenge with VLPs. Statistical significance was determined by Mann–Whitney test. *P<0.05, **P<0.01, ***P<0.001. Data are representative of three independent experiments.

The reactivation requirements of memory B cells, particularly the need for T-cell help, are not fully understood. However, reactivation of memory B cells by antigen, especially viruses, can occur independently of T cells^24^,^25^,^26^,^27^, yet seems to require specific niches in secondary lymphoid tissue^28^. To test whether ITT signalling contributes to the improved reactivation of IgG-switched memory B cells in the absence of T-cell help, we employed a cell transfer approach in which wild-type and YF-mutant mIgG1-expressing memory B cells were transferred into *Rag1*-deficient recipient mice. To be able to monitor the responsiveness of memory B cells expressing other Ig isotypes and to generate a competitive environment, we transferred total B cells depleted of T cells. The transferred cells were derived from animals that had been hyperimmunized with glycoprotein B of human cytomegalovirus (hCMV) (Fig. 7a) and were adjusted to contain equal numbers of glycoprotein B-specific mIgG1-positive cells (Supplementary Fig. 8). Consistent with our previous observation (Fig. 3b) the frequency of glycoprotein B-specific mIgG1-expressing cells 20 weeks after the last booster immunization was lower in ITT-mutant mice than in wild-type animals (Fig. 7b and Supplementary Fig. 8A). Therefore, we transferred roughly 1.5 times more B cells expressing other Ig isotypes from mIgG1-YF mice (Supplementary Fig. 8B). Subsequently, recipient animals were challenged with virus-like particles that contained glycoprotein B in their envelope, which triggers memory B-cell reactivation but does not generate a primary response^26^. During the entire course of the experiment, glycoprotein B-specific IgG1 titres were consistently three times higher in animals that had received wild-type mIgG1-expressing B cells than in animals that had received ITT-mutant cells (Fig. 7c). As experimental control, we measured the simultaneous production of IgG2c antibodies against glycoprotein B, which was enhanced in recipients that had received B cells from mIgG1-YF-mutant mice (Fig. 7d). Since we transferred slightly more cells from ITT-mutant mice to obtain equal numbers of mIgG1-expressing cells, the enhanced IgG2c production might be due to a higher number of transferred IgG2c-positive cells and/or compensatory mechanisms as observed before (Fig. 3 and^19^). At any rate, the results show that ITT-mediated improved reactivation of mIgG-positive memory B cells occurs also in the absence of T-cell help and thus is a B-cell-intrinsic feature.

## Discussion

Immunoglobulin class-switch recombination alters the molecular composition of BCRs. While the canonical Igα/β signalling unit remains unchanged, mIgG- (and mIgE-) BCRs are equipped with two copies of an additional tyrosine-based signalling motif referred to as ITT. In this study, we demonstrate that the ITT is a key determinant of humoral immunological memory *in vivo*. This provides a molecular explanation for the phenotype of a number of mouse models in which the entire cytoplasmic tails of mIgG1 or mIgE were either deleted or transferred to mIgM, which indicated their importance for the production of IgG and IgE antibodies^19^,^20^,^21^. Furthermore, B cells of ‘mIgG1-only' mice exhibit enhanced BCR signalling and a prolonged lifespan compared with mIgM-expressing cells^23^. Here we show that the biological activity of cytoplasmic mIgG tails is mainly mediated by the ITT signalling motif.

Antigen-induced phosphorylation of the ITT creates a docking site for the versatile adaptor protein Grb2, the recruitment of which amplifies BCR-proximal signalling events as well as BCR-induced cell proliferation^16^,^17^. Consistently, B-cell-specific inactivation of the *Grb2* gene in the mouse impairs reactivation of IgG-switched memory B cells, corroborating the importance of the ITT–Grb2 interaction for efficient antibody recall responses^17^,^29^. The most salient signalling effect of ITT-mediated Grb2-recruitment into the BCR signalosome is the enhanced activation of phospholipase C-γ2 (PLC-γ2), concomitant with a greatly prolonged influx of Ca^2+^ across the plasma membrane. In line with this, homoeostasis of B-cell memory relies on the expression of PLC-γ2 since its cell-type-specific ablation in mIgG1-expressing B cells causes reduced formation and survival of IgG1-switched memory B cells^30^. Furthermore, in B cells the phosphatase calcineurin, which controls the activation of transcription factor NF-AT, is specifically required for terminal differentiation into plasma cells^31^. Considering that the activity of calcineurin is stimulated by Ca^2+^/calmodulin it appears possible that ITT-mediated prolongation of mIgG–BCR-induced Ca^2+^ mobilization augments the activity of calcineurin thereby supporting the differentiation of IgG-switched B cells into plasma cells.

Plasma cell differentiation is generally considered to be governed by two antagonizing groups of transcriptional regulators that either maintain the mature B-cell phenotype, such as Pax5 and Bcl-6, or induce the plasma cell differentiation programme like Irf4 and Blimp-1 (ref. 32). Expression of either set represses the other one and elimination of Bcl-6 and Pax5 expression seem prerequisite for plasma cell differentiation to occur. Signals from the BCR might tip the balance between these two sets of transcription factors in favour of the plasma cell differentiation programme in several ways. First, BCR-induced proteasomal degradation of Bcl-6 has been reported to occur in a MAP kinase-dependent manner^33^. Second, in a reciprocal way expression of Irf4 is induced on BCR stimulation^34^,^35^. Third, the transcription factor Stat3, which acts in concert with Irf4 to induce expression of Blimp1 (ref. 36), is activated on BCR stimulation^37^,^38^. Thus, ITT-mediated enhanced signalling of mIgG–BCRs may facilitate degradation of Bcl-6 and/or influence the activity of other components that govern plasma cell differentiation such as Irf4 and Stat3. Consistent with such a scenario, B-cell-specific deletion of *stat3* results in a selective deficiency of IgG-producing plasma cells despite normal formation of germinal centres and memory B cells^39^. Besides improved BCR signalling, differential gene expression between memory and naive B cells has been reported and suggested to be involved in improved reactivation of memory B cells^40^,^41^,^42^,^43^. Furthermore, it has been proposed recently that the ability of both mIgM- and mIgG-expressing memory B cells to generate antibody-secreting cells on antigen challenge is primarily determined by their maturation stage that is reflected by expression of the cell surface receptors PD-L2 and CD80 (ref. 43). However, this conclusion was based on cell transfer experiments that did not reflect a physiological environment in which each (memory) B cell has to compete for antigen with antibodies as well as with antigen-specific (memory) B cells of other Ig isotypes that stem from the primary response^44^,^45^. Our data and that of other groups clearly show that the responsiveness of memory B cells is under control of the mIg isotype integrated into the BCR^19^,^21^,^45^,^46^.

Consistently, previous *in vivo* studies showed that a cytoplasmic mIgG tail improves antigen-dependent as well as antigen-independent survival of B cells in the mouse^21^,^23^. In line with these earlier observations, inactivation of the ITT compromised the maintenance of IgG1-switched cells in our experiments. Even though we cannot say whether this was due to diminished homoeostatic proliferation, impaired survival or altered migration of ITT-mutant cells, data from Waismann *et al*. suggest that an mIgG cytoplasmic domain improves the survival of B cells in an antigen-independent manner^23^. Hence, it appears likely that the ITT motif not only improves antigen-induced signalling but probably also antigen-independent tonic signalling of mIgG–BCRs.

In conclusion, our data show that the persistence and reactivation of IgG-switched B cells are under the control of the BCR and its cytoplasmic signalling machinery. The enhanced activation of this signalling machinery by the ITT may provide a clue as to why mIgG-expressing memory B cells are preferentially reactivated over mIgM-expressing cells in secondary immune responses^45^,^46^. Germinal centre-associated events, however, like generation of mIgG-expressing cells and antibody affinity maturation occur independently of ITT signals. In agreement with these findings, recent studies demonstrated that BCRs of germinal centre B cells are silenced by high phosphatase activity^47^ and that it is the effectiveness of antigen presentation to T helper cells that determines the fate of a B cell in a germinal centre reaction^48^,^49^,^50^. These observations may explain the dispensability of the ITT in germinal centre processes. Nevertheless, the phenotype of ITT-mutant mice reveals that the BCR on IgG isotype-switched cells is endowed with the intrinsic ability to promote humoral immunological memory even in the absence of cognate T-cell help.

## Methods

### Mice

mIgG1-YF knock-in mice were established using DNA from a C57BL/6 clone (RP23-331J13), in which the 19th codon of the transmembrane exon M2 of the γ1 immunoglobulin locus was mutated from TAC to TTC causing an amino acid exchange from Y to F. In addition, a neomycin resistance cassette with flanking loxP sites was inserted into the *Eco*RI restriction site between the two transmembrane exons M1 and M2 of the γ1 immunoglobulin locus. The targeting vector was electroporated into JM8.F6 ES cells (kindly provided by William C. Skarnes, Wellcome Trust Sanger Institute, Cambridge, UK), and recombinant ES cells were injected into eight-cell albino outbred embryos to generate chimeric offspring. After germline transmission, transgenic offspring were bred to EIIa-cre mice^51^ to delete the resistance cassette. Mice were held under specific pathogen-free conditions in individually ventilated cages and kept on a pure C57BL/6 background. Wild-type C57BL/6 mice used in Figs 2, 4 and 7 were purchased from Charles River (Sulzfeld, Germany). All other mice were bred in house. All animal experiments were approved by the state office for consumer protection and food safety of Lower Saxony and performed according to institutional and national guidelines.

### Immunizations

Cohorts of 8–12 weeks old age-matched mice were injected intraperitoneally with 50 μg OVA (Sigma-Aldrich, Taufkirchen, Germany) or 50 μg NP–KLH (Biosearch Technologies, Novato CA, USA), each with 25 μl Imject Alum (Thermo Fisher Scientific, Bonn, Germany), and boosted as indicated.

### Adoptive transfer of memory B cells

Transfer was performed as previously described^28^. Briefly, mice were immunized twice intraperitoneally with 5 μg glycoprotein B derived from hCMV (a gift from Sanofi Pasteur, Lyon, France) in Imject Alum at intervals of 5 weeks and with 5 μg glycoprotein B in PBS intravenously 5 weeks later and then rested for another 20 weeks. Subsequently, single-cell suspensions of splenocytes were depleted of T cells by complement lysis, followed by Ficoll gradient purification and enrichment of CD19+ B cells by magnetic cell sorting (Miltenyi Biotec). For each donor the frequency of glycoprotein B-specific IgG1+ memory B cells was determined by FACS, and total CD19+ B lymphocytes adjusted to contain 200 glycoprotein B-specific IgG1+ memory B cells were transferred into one or two *Rag1*-deficient recipient mice. One week after transfer, the recipients were boosted with 0.5 μg glycoprotein B-containing hCMV-dense bodies (virus-like particles) as described by Weisel *et al*.^28^ and bled regularly.

### ELISA

Nunc-Immuno MaxiSorp plates were coated over night at 4 °C with either 1 mg ml^−1^ OVA (Sigma-Aldrich) or 10 μg ml^−1^ NP1-BSA or NP14-BSA (selfmade using 4-Hydroxy-3-nitrophenylacetic acid active ester, Biosearch Technologies) in 50 mM carbonate-bicarbonate buffer. Sera were diluted in PBS with 1% BSA and incubated over night at 4 °C. Antigen-specific antibodies were detected with goat anti-IgG1-HRP (SouthernBiotech, catalogue no. 1071-05, dilution 1:1,000) or biotinylated anti-IgG1a (clone 10.9, BD Biosciences (BD) catalogue no. 553500, dilution 1:1,000) or anti-IgG1b (clone B68-2, BD catalogue no. 553533, dilution 1:1,000) followed by Neutravidin-HRP (Pierce, catalogue no. 31001, dilution 1:10,000). ELISAs were developed with ABTS (Sigma-Aldrich), read with PowerWave 340 (BioTek, Bad Friedrichshall, Germany) and analysed with Gen5 Software (BioTek). Total Ig concentrations were measured with the Clonotyping System-HRP (SouthernBiotech).

### ELISPOT

MultiScreen HTS filter plates (Millipore) were coated with either OVA or NP1-BSA, or NP14-BSA according to manufacturer's instructions. After incubation with 1–10 × 10^5^ cells for 4 h, captured antibodies were detected with the same detection antibodies and same dilutions as described for ELISAs and visualized with the AEC Chromogen Kit (Sigma-Aldrich). Wells were photographed with an ImmunoSpot analyzer (Cellular Technology Limited) and spots were either counted manually or using Bioreader software (BIO-SYS).

### Flow cytometry

Single-cell suspensions were prepared from bone marrow and spleen. Erythrocytes were removed by incubation with 0.15 M NH4Cl, 20 mM HEPES for 5 min at room temperature. Cells were then incubated with anti-mouse CD16/CD32 (Fc Block, clone 2.4G2, BD catalogue no. 553142, dilution 1:100) and stained for 60 min on ice with fluorochrome-conjugated mAbs against CD38 (clone 90, PacificBlue, BioLegend catalogue no. 102720, dilution 1:500), IgG1 (clone A85-1, FITC, BD catalogue no. 553443, dilution 1:500), B220 (clone RA-3-6B2, PECy7, BD catalogue no. 552772, dilution 1:500), NP(8)-PE (Biosearch Technologies, catalogue no. N-5070-1, dilution 1:100) and glycoprotein B-Cy5 (selfmade). A cocktail of biotinylated antibodies against CD3ɛ (145-2C11, BioLegend catalogue no. 100304, dilution 1:500), CD49b (DX5, BioLegend catalogue no. 108904, dilution 1:500), F4/80 (BM8, BioLegend catalogue no. 123106, dilution 1:500), TER-119 (TER-119, BioLegend catalogue no. 116204, dilution 1:500) and Ly6G/C (RB6-8C5, BioLegend catalogue no. 108404, dilution 1:500) in combination with APC-conjugated streptavidin (BD catalogue no. 554067, dilution 1:500) was used as negative marker. For intracellular phosflow stainings splenic B cells were isolated by magnetic bead cell sorting (B-cell isolation kit, Miltenyi Biotech, order no. 130-090-862) following stimulation of the cells with either anti-IgG or anti-IgM F(ab')_2_ fragments (Jackson Immuno Research, catalogue no. 115-006-071 and 115-006-075, respectively) using the indicated concentrations for 5 min at 37 °C in RPMI 1640 without serum. Cells were spun down, resuspended in BD Phosflow Perm/Wash Buffer I (BD) and stained with Alexa Fluor 647-conjugated antibodies to either phospho-SLP65 (pY84, clone J117-1278, BD catalogue no. 558443, dilution 1:6) or phospho-Akt (Ser473, clone D9E, BD catalogue no. 560343, dilution 1:6) and FITC-conjugated anti-IgG1 (clone A85-1, BD catalogue no. 553443, dilution 1:500). Stained cells were examined on a LSRII flow cytometer (BD Biosciences), and data were analysed with the FlowJo software (Tree Star, Inc.). Viable lymphocytes were gated as judged by forward/sideward scattering in all experiments.

### Ca^2+^ measurements

Splenocytes from three age-matched male mice immunized with sheep red blood cells for 11 days were pooled. B cells were enriched by magnetic CD43-depletion (Miltenyi Biotech) and stained with rat anti-mouse IgG1-FITC Fab fragments (clone A85-1, BD catalogue no. 553443). Fab fragments were generated by papain cleavage using the Pierce Fab Micro Preparation Kit (Thermo Scientific) according to the manufacturer's instructions. Subsequently, cells were loaded with Indo-1-AM as previously described^16^. mIgG–BCRs were stimulated with 20 μg ml^−1^ goat anti-mouse IgG F(ab')_2_ fragments (Jackson Immuno Research). For analysis of mIgG1-expressing cells, FITC-positive cells were gated.

## Additional information

**How to cite this article:** Lutz, J. *et al*. Reactivation of IgG-switched memory B cells by BCR-intrinsic signal amplification promotes IgG antibody production. *Nat. Commun.* 6:8575 doi: 10.1038/ncomms9575 (2015).

## Supplementary Material

Supplementary Information Supplementary Figures 1-8
