# Supplementary material for: Reactivation of IgG-switched memory B cells by BCR-intrinsic signal amplification promotes IgG antibody production
Source: Nat Commun. 2015 Oct 13;6:8575. doi: 10.1038/ncomms9575 (PMC4633962; doi:10.1038/ncomms9575)
Supplement: Supplementary Information — Supplementary Figures 1-8 [file ncomms9575-s1.pdf]

A

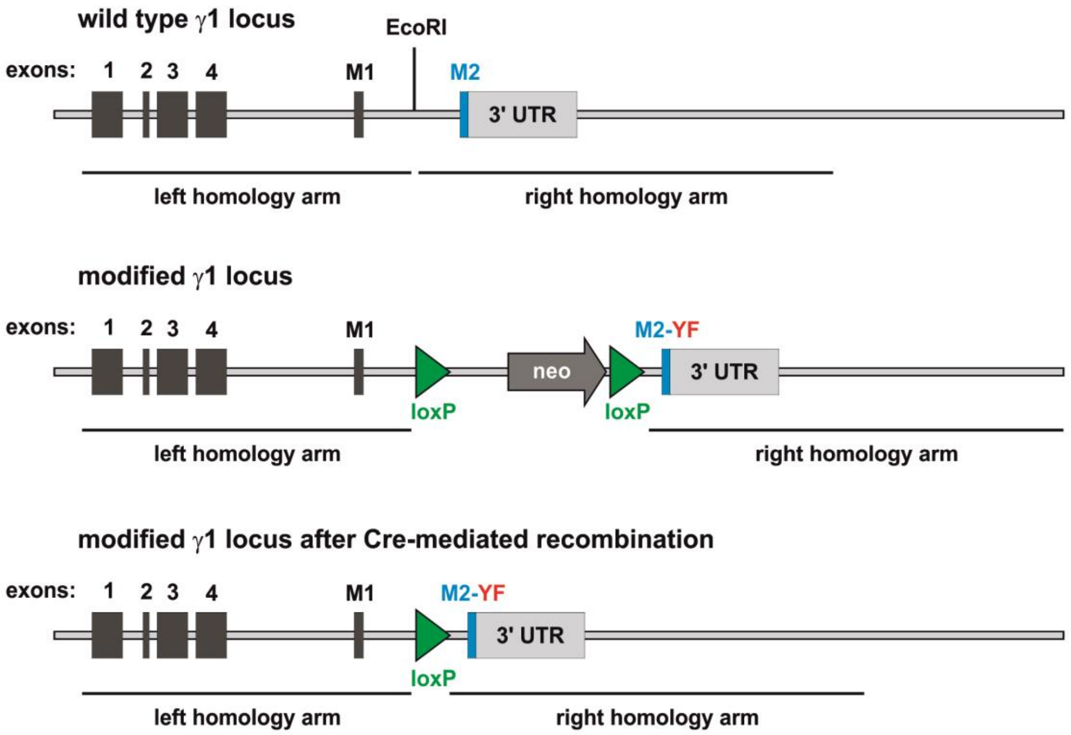

B

gtccccaactctctccctgcaccatttgtcaactgtcccctgaatt***GCTAGC****ATAACTTCGTATAATGTATGCTATACGAAG*
*TTAT****AAGCTTGCGGCCGGCCGCCACCTCGAGCATGCATCTAGTAGTTGTCTACAAAGAGACTCCAAGGGCGAATT***cccatgt
tgttcccacactgttagtgtgtaatgtgctctgtcccaggtgtaccttgttccgtgctgtctcacttcatcgcccattctgt
ccttgtaccaaccccactctatcaccacactgtccctatgcactgcccacattgtcctcatactgtcccattttgtatcttc
atcctgtccccatagtgtccaatgatctaccccacactattcccacttcatgcccctacaatttccctattccattcctctc
tggtcaccatgccatccttcccactcctgcacagctggagagggactcccgggatgagtccttgcccagatgagctacctat
ctagaggagtcttcaggtgggaagggaatgcagtcttgatcttggtcttattcaccctgtctcacag**GTAAAGTGGATCTTC**
TCCTCGGTGGTGGAGCTGAAGCAGACACTGGTTCCTGAATTCAAGAACATGATTGGGCAAGCACCCTAGgccacctcctgta
atggcatttcccaggccccgaaggac

inserted nucleotides
loxP site
EcoRI site
M2 exon
YF mutation

C

γ1m YF:

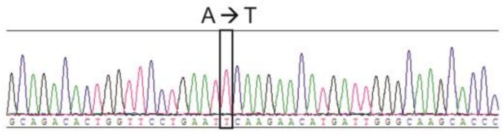

D

mouse γ1m wt KVKWIFSSVVELKQTLVPEYKNMIGQAP

mouse γ1m YF KVKWIFSSVVELKQTLVPEFKNMIGQAP

Supplementary Figure 1 Generation of mIgG1-YF mice. (**A**) Targeting strategy. Upper panel: schematic organization of the murine ɣ1 immunoglobulin locus. The EcoRI restriction site between exons M1 and M2 was used to insert a loxP site-flanked neomycin resistance cassette into the targeting vector. A point mutation in the ɣ1-M2 exon leading to a tyrosine-

to-phenylalanine (YF) substitution in the cytoplasmic domain of membrane-bound ɣ1 was located in the right arm of homology. The targeting vector was introduced into JM8.F6 ES cells by homologous recombination (middle panel). The loxP site-flanked neomcyin resistance cassette was deleted by breeding germline transmitted mice with the EIIa-Cre deleter strain (lower panel). (**B**) Genomic DNA sequence of targeted region. Nucleotides that were introduced into the mouse genome by gene targeting are indicated in upper case italics. The remaining loxP site is indicated in green. The coding region of the manipulated M2 exon is depicted in blue, the introduced point mutation in red. (**C**) Sequencing chromatogram showing the ITT-inactivating mutation in the mouse genome. (**D**) Amino acid sequences of wild type and ITT YF-mutant cytoplasmic domains of mouse ɣ1m.

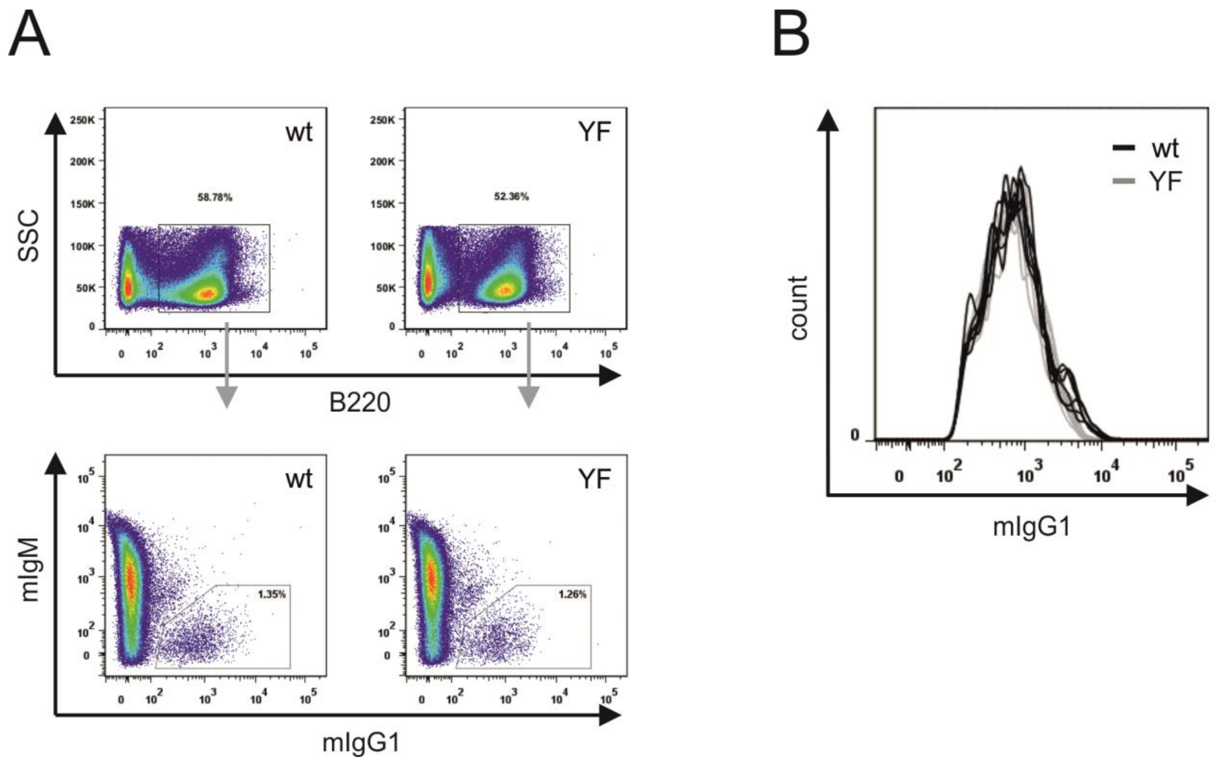

Supplementary Figure 2 Surface expression of wild type and ITT-mutant mIgG1-BCRs. Wild type (wt) and ITT-mutant mIgG1-YF (YF) mice were immunized with sheep red blood cells and analyzed on day 12. (**A**) Gating strategy. B220-positive live splenic lymphocytes were analyzed for expression of mIgM and mIgG1. (**B**) Histogram overlay of seven individual animals per genotype. In some experiments, we observed a subtle reduction in surface expression of YF-mutant mIgG1 in the order of 20 – 30%. However, such small differences did not have detectable effects on the signaling capacity of mIgG-BCRs in transfected cells.

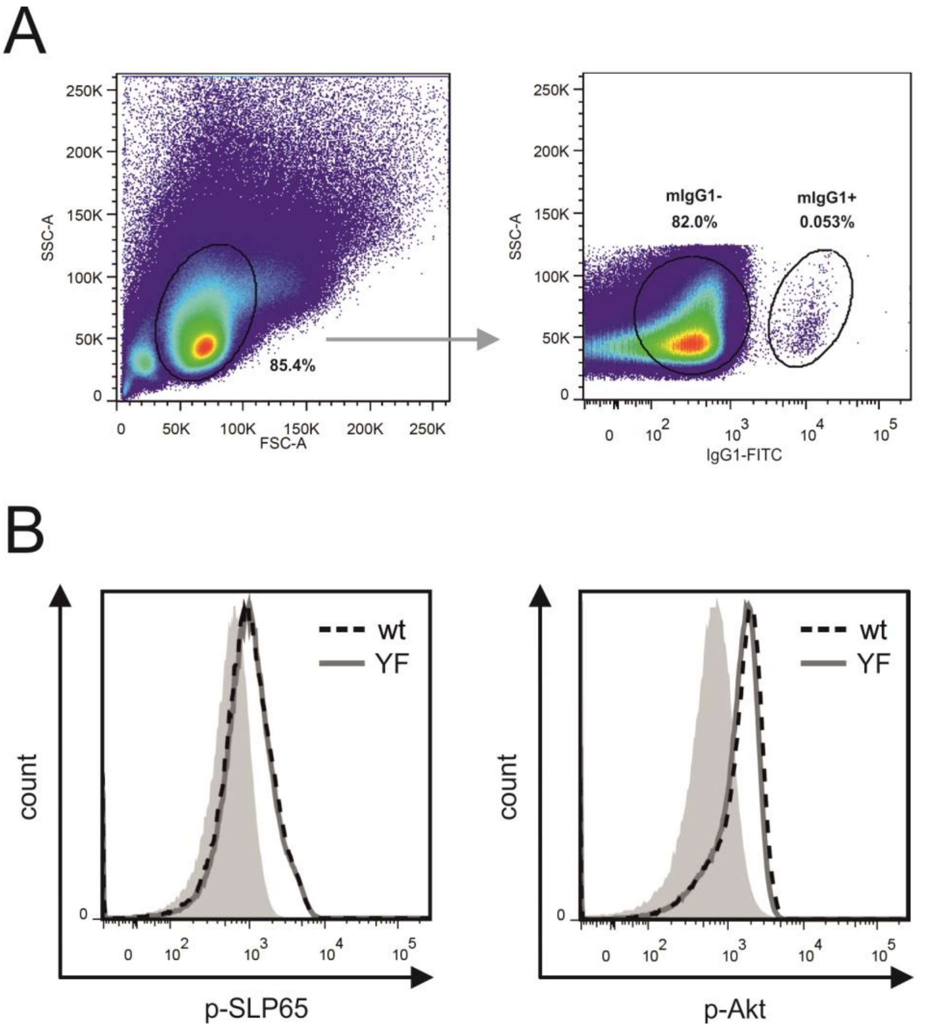

Supplementary Figure 3 Appendix to figures 1A and 1B. (**A**) Gating strategy for cells shown in figures 1A, 1B and supplementary figure 3B. (**B**) Phosflow analysis of phospho-SLP65 (p-SLP65) (left panel) and phospho-Akt (p-Akt) (right panel) in mIgG1-negative splenic B cells from wild type (wt, dashed black lines) and mIgG1-YF (YF, grey lines) mice stimulated with 10 µg per ml anti-IgM F(abˈ)$_2$ fragments for five minutes. Unstimulated control cells are shown as filled histograms.

A

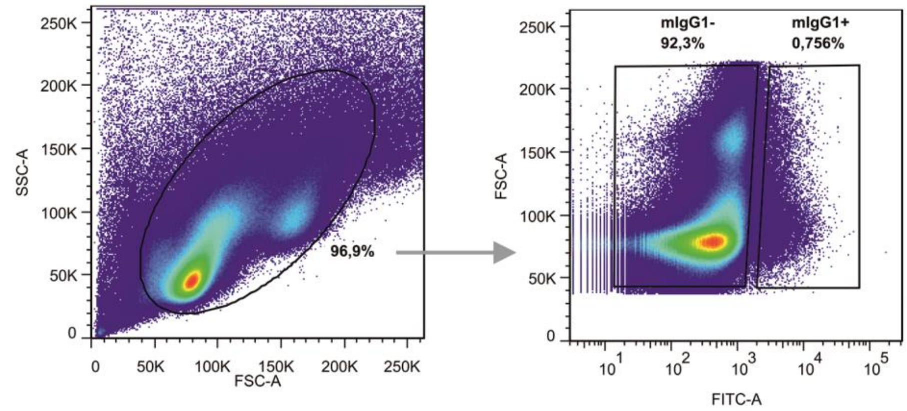

B

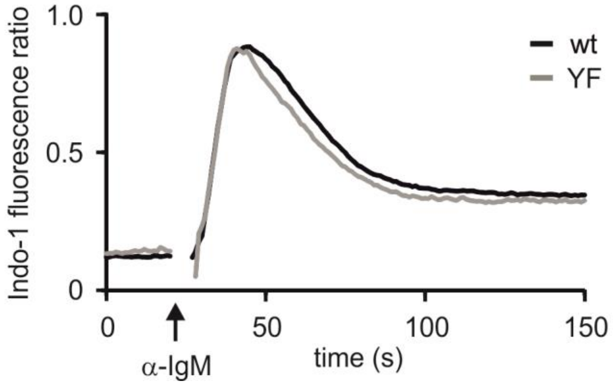

Supplementary Figure 4 Appendix to figure 1C. (**A**) Gating strategy for cells shown in figure 1C and supplementary figure 4B. Splenic B cells from age-matched male mice of each genotype that had been immunized with sheep red blood cells for 11 days were enriched by depletion of non-B cells with anti-CD43 magnetic microbeads (Miltenyi Biotec). B cells from three spleens of each genotype were pooled, loaded with Indo-1-AM and stained with FITC-labeled monomeric Fab fragments against IgG1. Ca^{2+} flux of mIgG1-positive cells is shown in figure 1C. (**B**) Ca^{2+} mobilization of mIgG1-negative cells was monitored before and after stimulation with anti-IgM $F(ab')_2$ fragments (20 µg per ml, indicated by an upward arrow) in the presence of 1 mM extracellular $CaCl_2$. wt: wild type (black curve), YF: mIgG1-YF (grey curve).

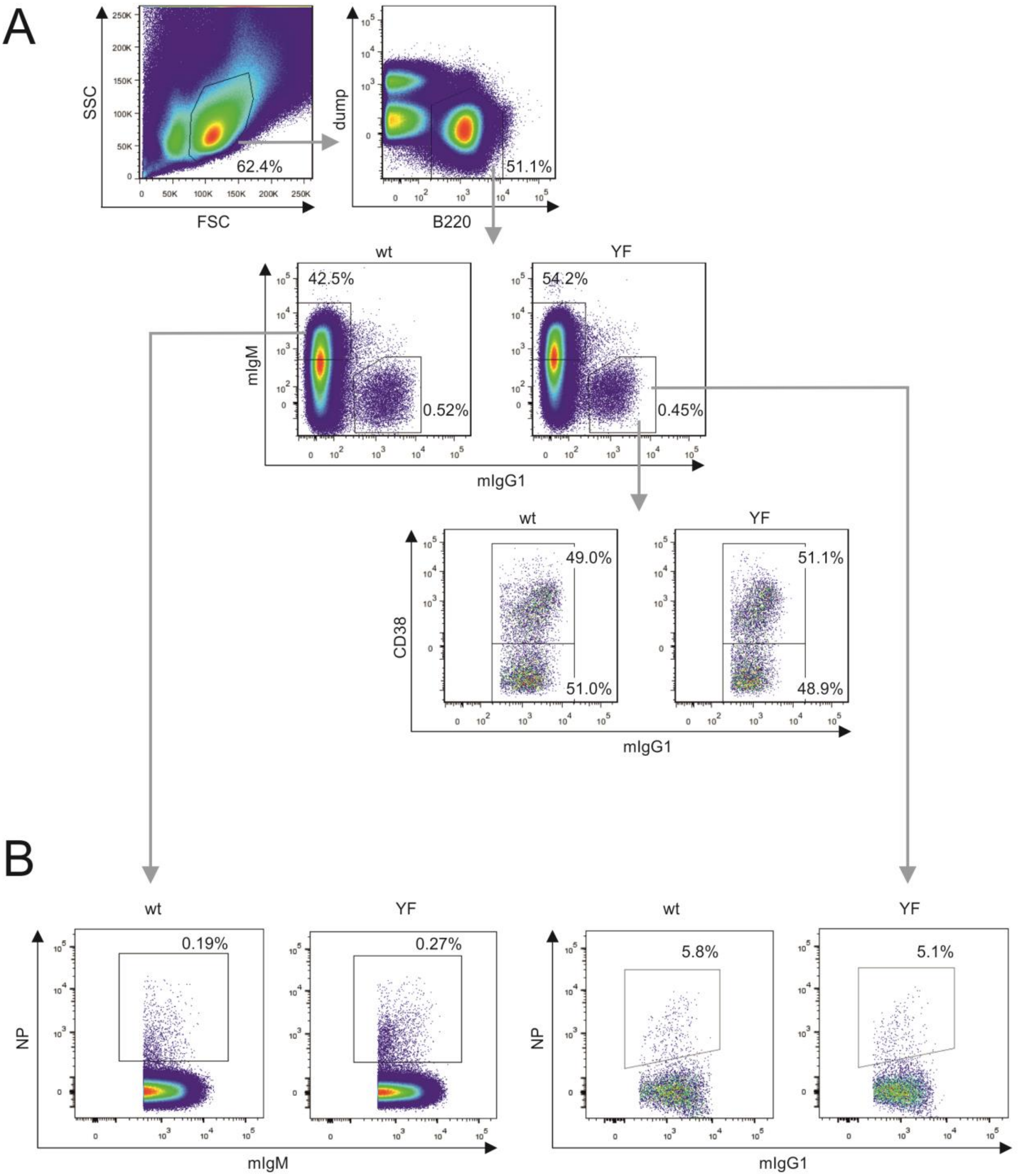

Supplementary Figure 5 Gating strategies for cells shown in figure 2 and supplementary figure 6. (**A**) Expression of CD38 on either mIgM-positive or mIgG1-positive splenic B cells of wild type (wt) and ITT-mutant (YF) mice 9 days after immunization with NP-KLH. Only cells staining positive for B220 and negative for the dump channel markers CD3ε, CD49b, F4/80, TER-119, and Ly6G/C were analyzed. (**B**) Gating strategy for analysis of NP-reactivity with NP8-PE.

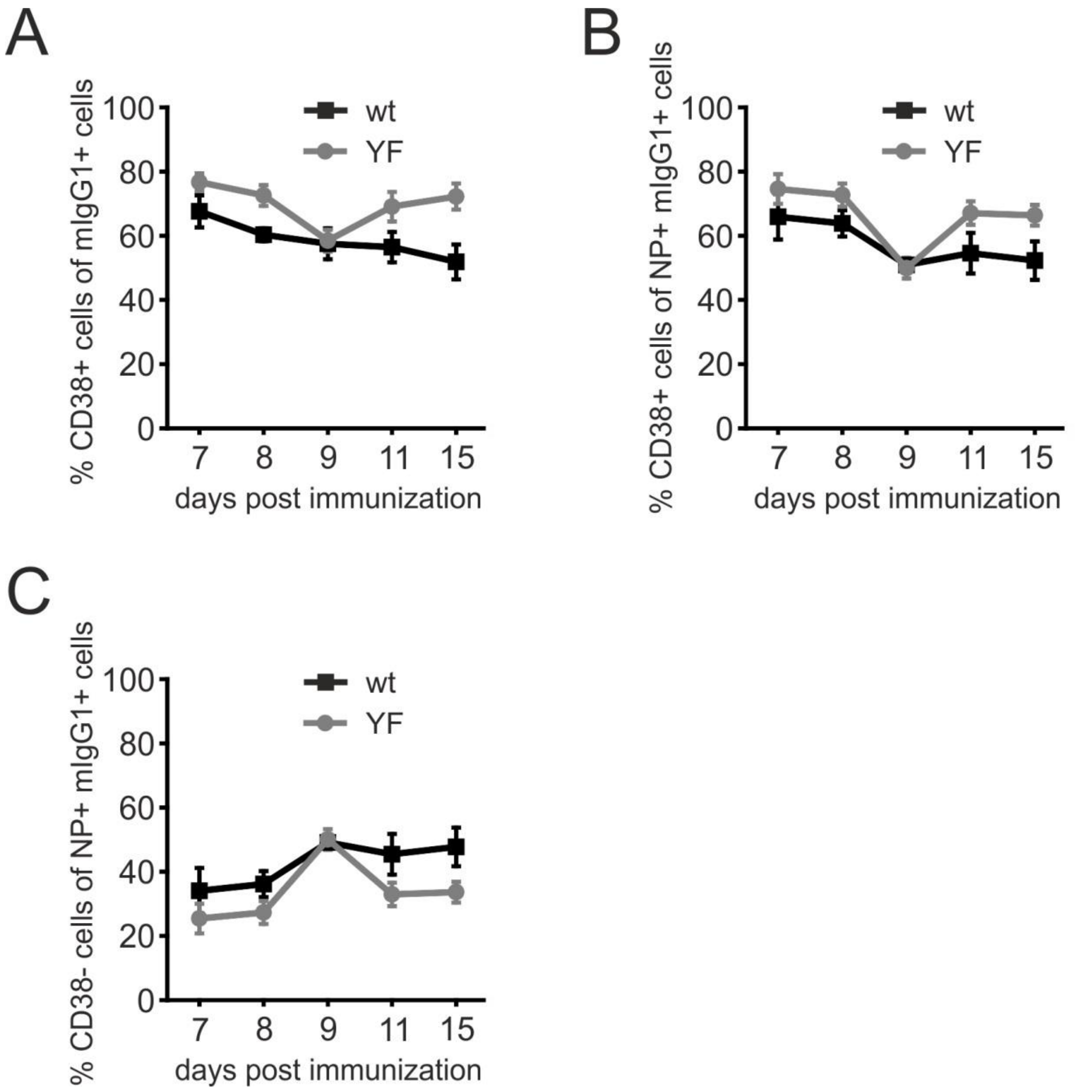

Supplementary Figure 6 Appendix to figure 2. Cells shown in figure 2 A-C were further analyzed. (**A**) Kinetics of CD38-positive, mIgG1-positive cells. (**B** & **C**) Kinetics of NP-reactive, mIgG1-positive cells expressing CD38 (**B**) or lacking CD38 (**C**).

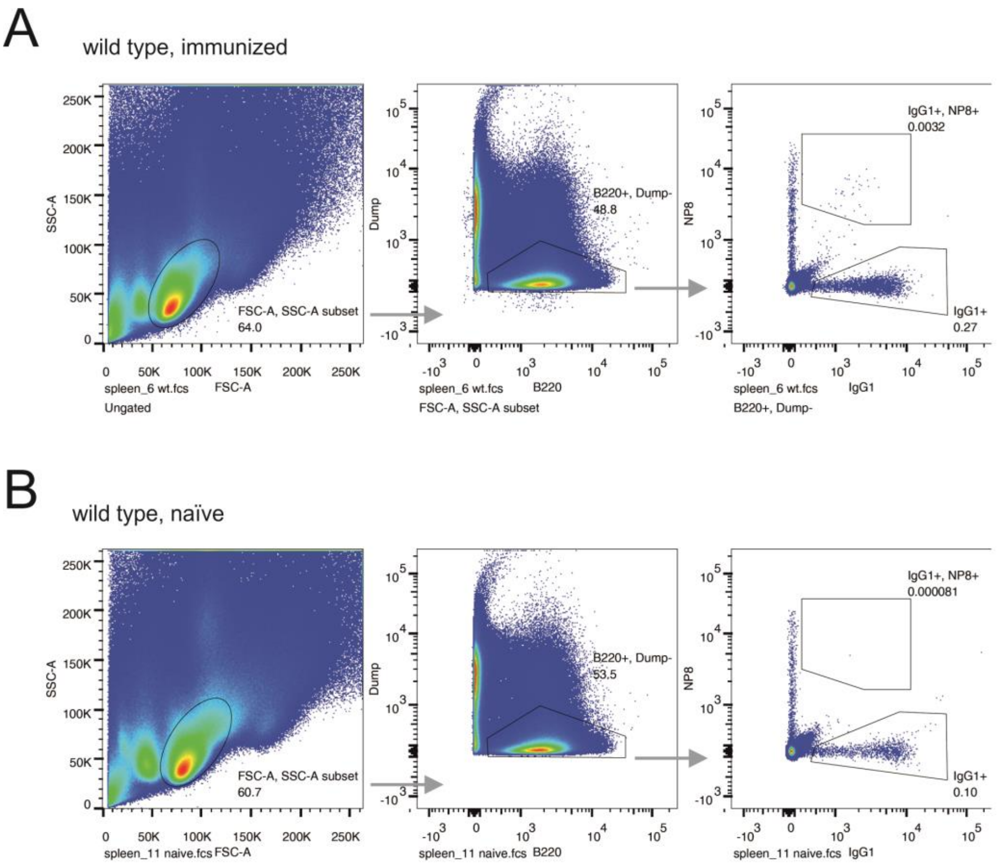

Supplementary Figure 7 Gating strategy for cells shown in figure 3. Only cells staining positive for B220 and negative for the dump channel markers CD3, CD49b, F4/80, TER 119, and Ly6G/C were analyzed. (**A**) Detection of NP-specific mIgG1-positive cells after immunization with NP-KLH. (**B**) Naïve mouse is shown as staining control.

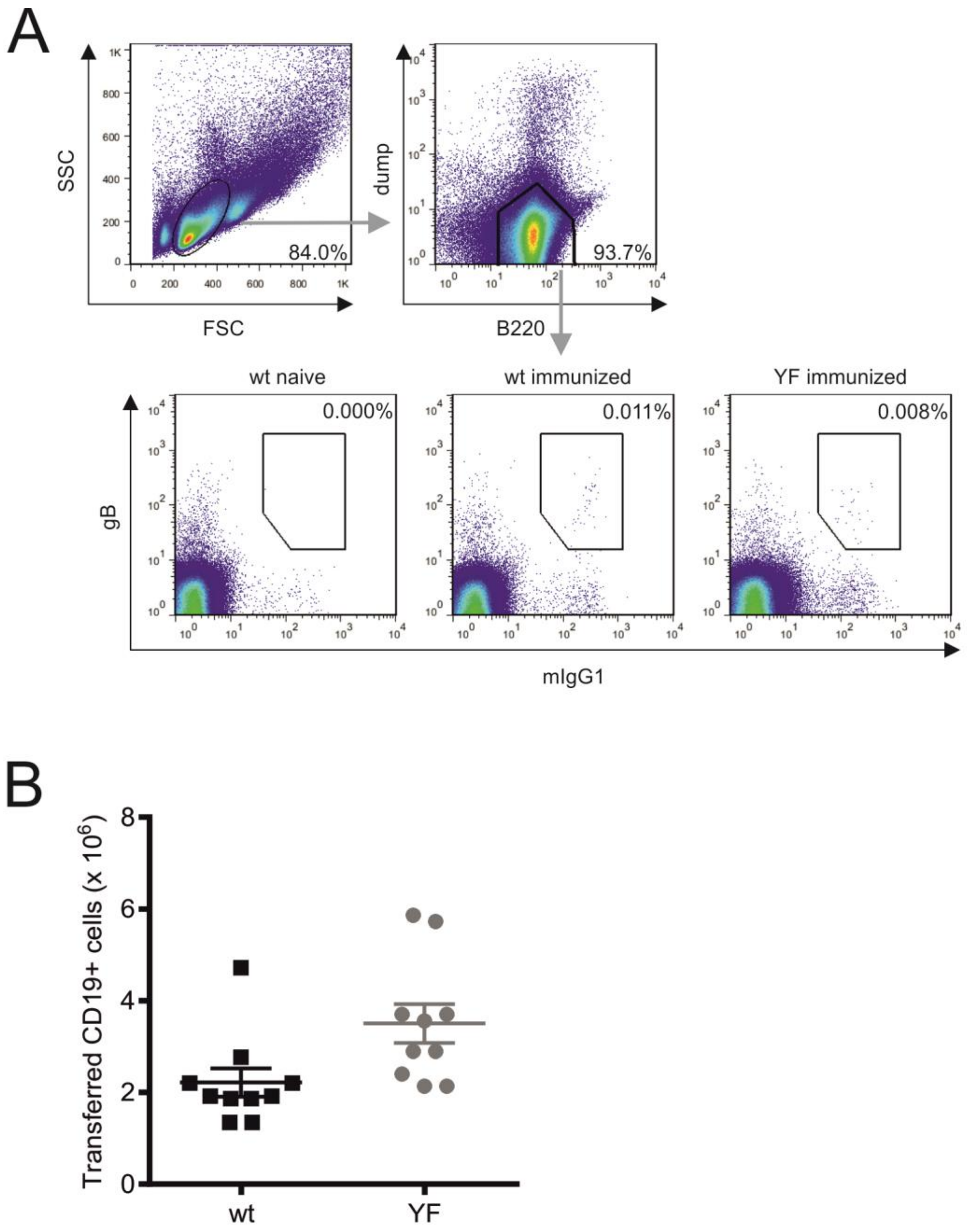

Supplementary Figure 8 Appendix to figure 7. (**A**) Analysis of the CD19-enriched, T cell-depleted splenocyte preparations from wild type (wt) and ITT-mutant (YF) mice that were used in cell transfer experiments. Frequencies of gB-specific mIgG1-positive, B220-positive, dump (CD3ε, CD49b, F4/80, TER-119, Ly6G/C)-negative memory B cells are indicated. Naïve mouse is shown as staining control. (**B**) Numbers of CD19-positive cells that were transferred into *Rag1*-deficient mice are shown for each recipient animal. Bars indicate mean ± SEM.
